# Supplementary material for: Interstitial Lung Disease Associated with Lung Cancer: A Case–Control Study
Source: J Clin Med. 2020 Mar 5;9(3):700. doi: 10.3390/jcm9030700 (PMC7141363; doi:10.3390/jcm9030700)
Supplement: Supplementary file 1 [file jcm-09-00700-s001.pdf]

Supplementary material: Silva Scoring System

Interstitial abnormalities (ground-glass opacities, consolidation, reticulation, honeycombing) are evaluated with a semi-quantitative scoring system.

The lung was divided into three zones: upper zone above the level of the tracheal carina; middle zone between the levels of the tracheal carina and the inferior pulmonary veins; and lower zone below the level of the inferior pulmonary veins. The zonal scores were added and divided by 3 to obtain an averaged lung score.

Grade 0: when these abnormalities are absent.

Grade 1: when the extent is between 1% and 4% of the lung parenchyma.

Grade 2: when the extent is between 5% and 25% of the lung parenchyma.

Grade 3: when the extent is between 26% and 50% of the lung parenchyma.

Grade 4: when the extent exceeds 50%.

Table S1. Treatment Options for Non-Small–Cell Lung Cancer (NSCLC) According to Stage

	NSCLC–ILD			NSCLC–noILD		
		Standard of care			Standard of care	
Stage	n	Yes	No	n	Yes	No
I	7	6, surgery<br>1, radiotherapy		19	19, surgery	
II	4	3, surgery	1, chemotherapy	14	1, surgery<br>11, surgery + chemotherapy	2, ChRT
IIIA	5	2, surgery + chemotherapy<br>1, ChRT	1, chemotherapy<br>1, radiotherapy	17	2, surgery + chemotherapy<br>7, ChRT<br>7, BSC	1, chemotherapy
IIIB	4	3, ChRT	1, chemotherapy	10	6, ChRT<br>2, BSC	1, chemotherapy<br>1, radiotherapy
IV	21	15, chemotherapy<br>2, surgery + chemotherapy<br>4, BSC		62	52, chemotherapy<br>1, surgery + chemotherapy<br>9, BSC	

LC, lung cancer; ILD, interstitial lung disease; ChRT, chemoradiotherapy; BSC, best supportive care.

Table S2. Treatment Options for Small-Cell Lung Cancer According to Stage

Stage	SCLC–ILD n	SCLC–ILD Standard of care: Yes	SCLC–ILD Standard of care: No	SCLC–noILD n	SCLC–noILD Standard of care: Yes	SCLC–noILD Standard of care: No
Limited	4	1, ChRT	3, chemotherapy	12	4, ChRT<br>3, surgery + chemotherapy	5, chemotherapy
Extensive	4	4, chemotherapy		12	12, chemotherapy	

LC, lung cancer; ILD, interstitial lung disease; ChRT: chemoradiotherapy.

Table S3. Patients Treated with Surgery

		LC–ILD N = 12	LC–noILD N = 41
LC Stage			
Localized			
	Alone	7	27
	With adjuvant chemotherapy	3	11
Locally advanced			
	After neoadjuvant chemotherapy	2	2
	After chemoradiotherapy		1
Surgical procedure			
	Wedge	1	1
	Segmentectomy	0	4
	Lobectomy	8	27
	Bilobectomy	1	2
	Pneumonectomy	2	7
Complications			
	Total	5	11
	Pulmonary infection	3	7
	Pulmonary non-infectious	0	2
	Cardiovascular	1	3
	Acute exacerbation	1	0
	Deaths	2	2

LC, lung cancer; ILD, interstitial lung disease.

Table S4. Patients Treated with Radiotherapy

			LC–ILD N = 8	LC–noILD N = 25
LC stage				
	Localized			
		Conformal radiotherapy	1	3
		Stereotactic radiotherapy	1	1
		Adjuvant radiotherapy	1	0
	Locally advanced			
		Chemoradiotherapy	5	21
Complications		Total	4	10
		Pulmonary infection	0	3
		Chemotherapy-related	2	5
		Radiation pneumonitis	2	3
		Deaths	0	2

LC, lung cancer; ILD, interstitial lung disease.
